# COVID-19–associated mucormycosis: Evidence-based critical review of an emerging infection burden during the pandemic’s second wave in India

**DOI:** 10.1371/journal.pntd.0009921

**Published:** 2021-11-18

**Authors:** Jesil Mathew Aranjani, Atulya Manuel, Habeeb Ibrahim Abdul Razack, Sam T. Mathew

**Affiliations:** 1 Manipal College of Pharmaceutical Sciences, Manipal Academy of Higher Education, Manipal, Karnataka, India; 2 CCS National Institute of Animal Health, Baghpat, Uttar Pradesh, India; 3 College of Medicine, King Saud University, Riyadh, Saudi Arabia; 4 Faculty of Medicine & Health Sciences, Universiti Putra Malaysia, Serdang, Selangor, Malaysia; 5 Researcher & Medical Communications Expert, Bengaluru, Karnataka, India; The University of Hong Kong, HONG KONG

## Abstract

Coronavirus Disease 2019 (COVID-19), during the second wave in early 2021, has caused devastating chaos in India. As daily infection rates rise alarmingly, the number of severe cases has increased dramatically. The country has encountered health infrastructure inadequacy and excessive demand for hospital beds, drugs, vaccines, and oxygen. Adding more burden to such a challenging situation, mucormycosis, an invasive fungal infection, has seen a sudden surge in patients with COVID-19. The rhino-orbital-cerebral form is the most common type observed. In particular, approximately three-fourths of them had diabetes as predisposing comorbidity and received corticosteroids to treat COVID-19. Possible mechanisms may involve immune and inflammatory processes. Diabetes, when coupled with COVID-19–induced systemic immune change, tends to cause decreased immunity and an increased risk of secondary infections. Since comprehensive data on this fatal opportunistic infection are evolving against the backdrop of a major pandemic, prevention strategies primarily involve managing comorbid conditions in high-risk groups. The recommended treatment strategies primarily included surgical debridement and antifungal therapy using Amphotericin B and selected azoles. Several India-centric clinical guidelines have emerged to rightly diagnose the infection, characterise the clinical presentation, understand the pathogenesis involved, and track the disease course. Code Mucor is the most comprehensive one, which proposes a simple but reliable staging system for the rhino-orbital-cerebral form. A staging system has recently been proposed, and a dedicated registry has been started. In this critical review, we extensively analyse recent evidence and guidance on COVID-19–associated mucormycosis in India.

## Introduction

India reported 31 million cases of Coronavirus Disease 2019 (COVID-19) as of July 17, 2021, trailing only behind the United States (US) with about 3 million cases [1]. An estimated 448,000 COVID-19 deaths are projected in the country by November 1, 2021 [2]. The Ministry of Health and Family Welfare (Government of India [GoI]) regularly issues travel and health advisories, provides guidance and inspiration to the healthcare community, sets up pandemic management taskforces, establishes helplines, enables mhealth surveillance (Arogya Setu app), opens counselling centres, and creates awareness materials for the public [3]. Despite all these efforts, the second wave that began in early 2021 was very upsetting, as people struggled to hospitalise infected patients, access oxygen supply, and handle the bodies of deceased patients. The largest cities of North India witnessed queues of ambulances with critically ill patients and a shortage of cremation facilities in graveyards, leading to open cremation on street sidewalks [4–6]. Insufficient pandemic mitigation and superspreader mass gatherings of religious and political interests are believed to have caused the worst COVID-19 surge [7,8].

The common COVID-19 manifestations, as observed in a study of over 136,000 cases during the initial months of infection spread in India, included high fever (88%), dry cough (67.7%), fatigue (38.1%), dyspnoea (18.6%), and muscle pain (14.8%), while 7.1% patients were asymptomatic [9]. A very recent (July 15, 2021) study reported an international cohort (*n =* 3,762) spanning 56 countries with confirmed or suspected “Long COVID,” a condition with a majority recovered only after 35 weeks since onset. The authors assessed the prevalence of over 200 symptoms affecting 10 bodily systems and revealed that the mean number of symptoms per patient was 55.9 (±25.5) [10]. Researchers warn that the impact of COVID-19 has been greatly neglected as patients continue to develop multisystemic manifestations for more than 7 months [10]. COVID-19 deteriorates immune status of patients, paves the way for opportunistic and secondary infections, and worsens preexisting clinical conditions [10,11].

## The surge in COVID-19–associated mucormycosis

Across borders, patients with COVID-19 experience notorious fungal coinfections during or after weeks or months of recovery. COVID-19–associated mucormycosis (CAM) has been reported in many countries like Austria, Brazil, Egypt, France, India, Iran, Italy, and the US [11,12]. A recent systematic review observed that CAM constitutes 0.3% of COVID-19 coinfections [13]. Mucormycosis is not unfamiliar to India; the case rate before 2019 was almost 70 times higher than in developed countries. The disease prevalence in India is predicted to be 140 cases per million population [8,14]. While India faces tough times during the second wave of COVID-19, there is a recent unexpected surge in CAM cases. As a result, GoI has declared it a notifiable disease, while several state governments have declared it an epidemic [8]. Various control measures and guidance on procuring and allocating treatment drugs to all states were taken up quickly [15].

COVID-19–associated rhino-orbital-cerebral mucormycosis (CAROCM) is the most common type observed during the current epidemic, followed by the pulmonary form [16,17]. Prior to the COVID-19 pandemic, the mucormycosis mortality was 50%. However, during the current pandemic situation in India, it has increased to 85%, mainly due to crowded hospitals, unavailability of healthcare resources, overburdened healthcare workers, and poor diagnostic quality [18]. Notably, a recent systematic review of 7 global CAM cases observed 100% mortality, though 43% of them received antifungal monotherapy [19]. For India, a low doctor/patient ratio and practising by unqualified or nonregistered healthcare professionals are other key hurdles that the country faces to tackle CAM [20]. Lack of awareness and inadequate health infrastructure have introduced additional burden [16]. Globally, at least 6 systematic literature reviews are available on CAM, as of July 19, 2021. The majority of publications are from India (*n =* 4) [21–24]; the largest review has included 82 cases [24]. Besides, newer observational studies and case series have emerged. An Indian multicentric epidemiological study reported a 2-fold increase in mucormycosis cases year-over-year (2019 versus 2020). The CAM prevalence was 0.27% among hospitalised patients [25]. An in-press editorial from the Indian Journal of Medical Microbiology claims that a GoI website has mentioned more than 31,000 CAM cases by June 13, 2021 [16]. By July 2021, the case count reached 40,000 [26].

Extensive discussions and strategic actions are being promulgated from political, administrative, regulatory, and healthcare flagbearers during this unprecedented time as the country faces an epidemic within a ravaging pandemic. Recently, a staging system for CAROCM has been proposed, and a dedicated registry for CAM has been started. The present critical review, which stands out from the limited reviews available as of July 19, 2021, attempts to comprehensively analyse recent evidence on CAM pathogenesis and its potential risk factors and intends to throw light on several India-specific treatment guidelines. We searched leading databases like PubMed, Web of Science, Ovid, Scopus, and Google Scholar for scholarly literature on CAM surge in India published until July 19, 2021, in English using the relevant keywords (“mucor*” OR “zygomycosis;” “COVID” OR “SARS-CoV” OR “coronavirus;” and “India”). Besides, we conducted a Google web search for CAM and COVID-19 statistics and potentially relevant news reports published before the above data cutoff period. We also reviewed the official websites of professional societies like the “Indian Medical Association” and governmental portals like the “Directorate General of Health Services–India” before the said date for any essential treatment and management guidelines. Later in September 2021, we had modified the post-submission manuscript based on the peer review comments and updated it with the recent references suggested by the reviewers.

## Causes, characteristics, and diagnosis

Mucormycosis is caused by various saprophytic fungi in the order Mucorales, including *Rhizopus*, *Lichtheimia*, *Mucor*, and *Rhizomucor* species, and the first 3 species account for three-fourths of all cases [27]. It is an opportunistic infection affecting the lungs, skin, gut, rhino-cerebral areas, and central nervous system (CNS) and presents a clinical condition specific to the organ system. It may also be present in a disseminated form [28]; postmortem analysis in a few patients with disseminated disease revealed that they did not receive systemic or surgical treatment [19]. *Rhizopus arrhizus* (previously named *Rhizopus oryzae*) is the most common causative agent globally, although the fungi responsible for infection differ between geographies. In India, *Apophysomyces* is the second most familiar species causing mucormycosis [29]. *Apophysomyces elegans* and *Rhizopus homothallicus* are commonly reported fungi in this region, while other uncommon ones, including *Mucor irregularis* and *Thamnostylum lucknowense*, have also been observed. This deep fungal infection is wrongly termed a black fungus by local media and even by some learned academic researchers by confusing dematiaceous fungi with those that cause mucormycosis [30,31]. A unique form of isolated renal mucormycosis has also been reported in India [32,33].

Mucormycosis is not contagious. The entry of infective spores present in the environment into the human body is by inhaling, ingesting or direct inoculation through wounds, and germinating into angioinvasive hyphae [19,31]. The most extensive observational study reported on CAROCM in India, as of July 19, 2021, included 2,826 patients from 22 states. This research, named COSMIC, conducted jointly by the Oculoplastic Association of India and the Indian Journal of Opthalmology, described the epidemiological features and clinical characteristics along with details of predisposing factors for developing CAROCM and presenting various management strategies and their reported outcomes. The most common CAROCM symptoms observed in that study included orbital/facial pain (23%) and oedema (21%), vision loss (19%), ptosis (11%), and nasal congestion (9%); the primary signs included periocular/facial oedema (33%), vision loss (21%), proptosis (11%), and nasal discharge (10%) [34]. Pulmonary mucormycosis mimics the symptoms of COVID-19, such as pyrexia, cough, and dyspnea [18].

The initial features of CAROCM appear in the paranasal sinuses before extending to the orbit and the cranial cavity. CAROCM can be suspected in patients with signs and symptoms as observed in the COSMIC study, along with the presence of necrotic ulcers in the nasal cavity/palate and orbital apex syndrome and cavernous sinus syndrome [34,35]. Mucormycosis involving the ear may have the characteristics of otitis externa, and the usual antibacterial therapy does not resolve the otological symptoms [36]. Double- and triple-mutant variants of SARS-CoV-2 have emerged in India during the second wave of COVID-19, which are more dreadful and are doubted to play a mightier role in CAM surge [37,38]. A recent systematic review that compared CAM cases of India (*n =* 233) with that of other countries (*n* = 42) suggested that pulmonary or disseminated disease form was associated with increased death rate, and treatment (in combination form) could improve survival [39]. Another cohort study identified cerebral involvement, and a higher HbA1c (≥8) level could significantly predict patient survival [40]. Diagnostic confirmation can be obtained by (a) direct microscopy with slides mounted with potassium hydroxide, particularly when it is necessary to start therapy immediately; (b) fungal culture; (c) biopsy of the affected lesions with adequate precaution; (d) radiological (computed tomography of ostiomeatal complex and magnetic resonance imaging with contrast) examinations; and (e) matrix-assisted laser desorption/ionisation time of flight mass spectrometric analysis [35,41–45]. Florid sinusitis and bone erosion may not be identified on radiological examinations at an early stage; therefore, initial findings should not lead to missed diagnosis [42]. REBOVasC checklist may help radiologists not to miss any critical findings in the imaging examination [46]. It focuses on the rhinosinus, extrasinus, bones, orbital, vascular, and CNS areas for infection spread.

## How does mucormycosis affect patients with COVID-19?

There are 3 possible theories for CAM related to immune and inflammatory processes: (1) COVID-19 causes significant lymphopenia, resulting in a dramatic reduction in the availability of T cells (CD4+ and CD8+) and opening the entry gate for opportunistic fungal infections; (2) increased pro-inflammatory markers in patients with severe disease; and (3) pronounced damage of pulmonary tissues by COVID-19 aids the invasive fungi, especially the airborne ones or those that attack through the respiratory system [47,48]. Hyperferritinemia caused by excess interleukin-6 (IL-6) release and macrophage activation results in increased availability of free iron within cells. This, in turn, causes endothelial destruction and inflammation called endothelitis. In addition, the hepcidin mimetic action of the virus further induces ferritin independent of the inflammatory reaction [17]. Thus, COVID-19–induced immunosuppression increases the risk of opportunistic infection, damages the endothelium and alveoles, easing the port of fungal invasion, and increases the glucose level due to the acute diabetes-like state caused by pancreatic damage; increased ferritin level supports fungal growth, and increased body temperature is optimal for thermotolerant fungi. Another exciting hypothesis revolves around the dysregulation of angiotensin-converting enzyme 2 expression in various bodily organs, immunosuppression, and the creation of a microenvironment system that increases the risk of coinfection in COVID-19 [49]. Up-regulation of 78-kDa glucose-regulated protein (GRP78), a heat shock protein, in patients with COVID-19 (5 times more than controls) due to increased glucose and iron content caused by diabetic ketoacidosis (DKA) or induced by dexamethasone use also facilitates fungal entry. Besides, it promotes Mucorales’ pathogenicity and virulence [17,50].

## Identifying risk factors and implementing preventive measures

The systematic review of global CAM (7 cases) reported a male predominance (85.7%), and all patients showed severe disease conditions [19]. Importantly, the COSMIC study enrolled patients from January 2020, i.e., since the beginning of COVID-19 in India. However, the surge in CAM incidences has been observed during the second pandemic wave in 2021. The results identified diabetes as the primary predisposing factor. Another multicentre CAM epidemiological study conducted between September and December 2020 during the first wave has also reported the exact reason for such a steep hike occurred lately. Over 65% of CAM patients (187 of 287 mucormycosis cases) had uncontrolled diabetes [25] and are thus the most significant risk factor for CAM. The phagocytes of immunocompetent hosts have the potential of kill Mucorales spores by producing oxidative metabolites. Uncontrolled hyperglycemia due to DKA can change this immunologic response, leading to reduced granulocyte phagocytosis with a modified polymorphonuclear leukocyte response [51]. Besides, Mucorales possess a ketone reductase enzyme that thrives in high glucose conditions and DKA, resulting in a poor prognosis [52]. India, the home of 1.38 billion people as estimated for 2020 [53], reportedly has 77 million patients with diabetes as of 2018. Diabetes accounts for 3.1% of all deaths [54,55]. Diabetes, when coupled with COVID-19–induced systemic immune change, results in decreased immunity and an increased risk of secondary infections [51,56]. A recent systematic review of 15 studies had confirmed the role of diabetes in CAM [57]. Managing diabetes is thus crucial for controlling both COVID-19 and CAM [58,59].

Another critical risk factor is the inappropriate use of corticosteroids [60]; 71.4% of cases reported in a systematic review had received corticosteroids [19]. In particular, three-quarters of Indian patients in a study conducted during the first wave of COVID-19 received corticosteroids (with more than 60% receiving inappropriate therapy) [25]. In the initial period of the pandemic, the Indian authorities recommended a higher methylprednisolone dose (approximately 70 to 140 mg) for patients with severe and critical COVID-19. This could have affected more immunocompromised patients and made them vulnerable to fungal attack. Later, it was reduced to the World Health Organisation–suggested dose of 32 mg/day. Besides, even the nonsevere patients took corticosteroids out of fear without medical counselling [8,61]. Corticosteroids tend to cause lymphopenia and T lymphocyte dysregulation. They may also induce hyperglycaemia in patients with diabetes [62,63]. In a COVID Care Centre in Western India, 3.36% (32 of 953) patients experienced CAM, while 93% of them had prior corticosteroids exposure [64]. Tocilizumab, an anti-IL-6 receptor monoclonal antibody, is the only immunosuppressant mentioned in the Indian guidelines on the COVID-19 management algorithm for off-label use [65]. Increased use of tocilizumab in patients with COVID-19 for inflammation control can reduce patient immunity and pose a significant risk for contracting CAM [66–68]. The use of an antifungal agent, voriconazole, in Mucorales-related infections does not yield efficacy; instead, it can lead to breakthrough mucormycosis infection.

Similarly, inappropriate use of broad-spectrum antibiotics in patients with COVID-19 may also increase CAM risk [14]. Both broad-spectrum antibiotics and antifungal prophylaxis interrupt the healthy sinonasal microbiome, possibly enabling opportunistic infections. Besides, macrolide antibiotics such as azithromycin also hinder IL-6 and thus disrupt antimicrobial immune response [68]. Notably, a recent clinical study comparing patients with CAM and controls has suggested no clear evidence on the role of zinc supplementation in CAM pathogenesis [69].

Neutropenia, leading to reduced intact immunity, is a risk factor for mucormycosis; thus, immunocompetent individuals do not become affected by it [47]. The unhygienic handling of oxygen or inferior tubing, contaminated masks, an impure water source used in humidifiers, and the use of the same oxygen mask for more than 2 patients for a longer period are also reported to be the causes of concern [66,70]. However, the involvement of humidifier water as the source is debatable. It is noteworthy that the passage of oxygen makes humidifier water agitated, thus preventing Mucorales to produce spores [31]. Since there is increased use of industrial oxygen during this crisis, appropriate handling is crucial for patient safety [14].

**Fig 1:**
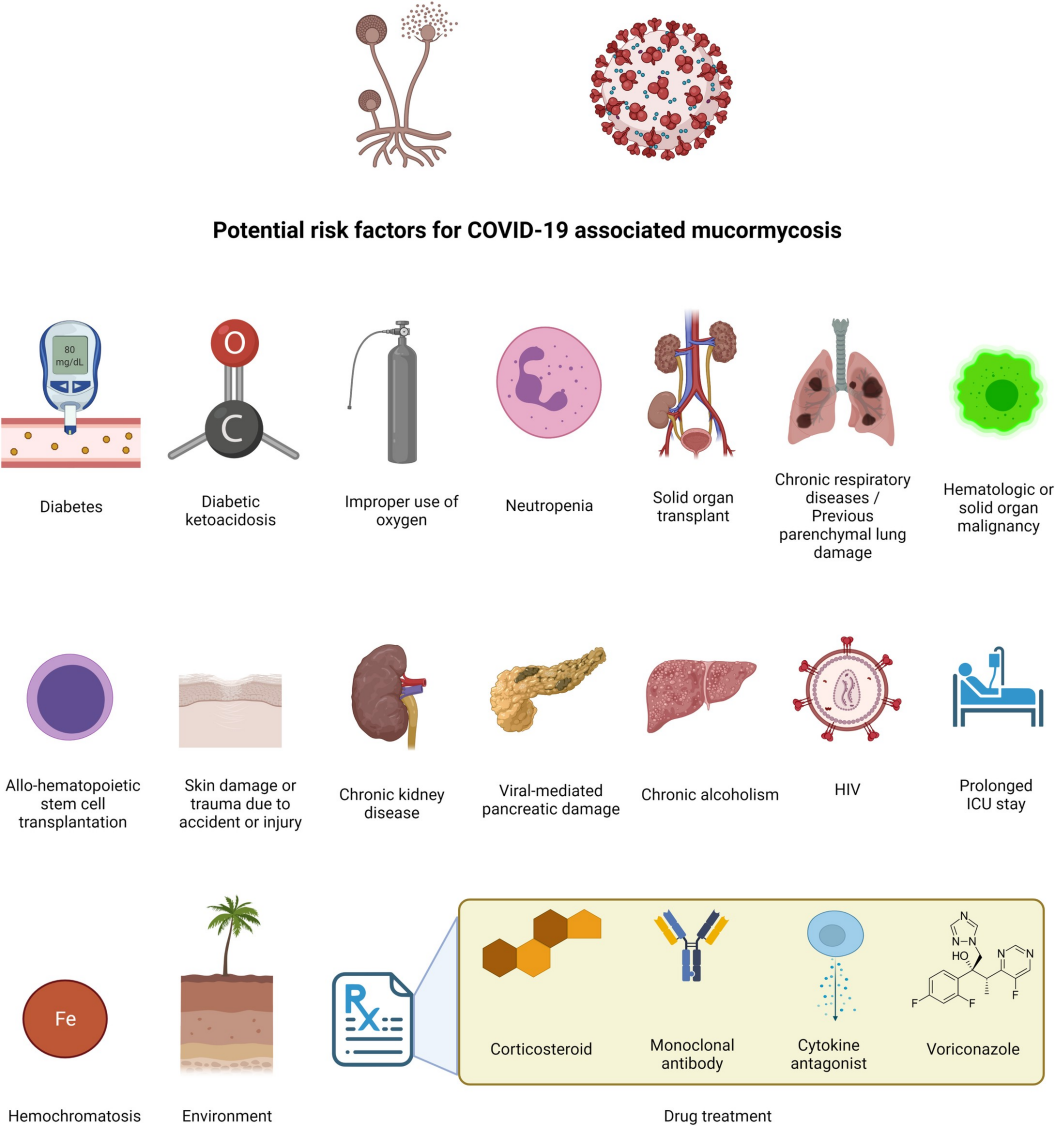
Potential risk factors for COVID-19–associated mucormycosis.

Albeit mucormycosis is rare in patients who received organ transplants (2 in 1,000 cases), retransplantation and chronic graft dysfunction are considered risk factors, mainly because of repeated immunosuppressive requirements [71,72]. Environmental factors, including humid climate (tropical/subtropical) and hot weather in several regions within India, may encourage fungal growth and be suitable for disease prevalence. Interestingly, periodic variations in mucormycosis have been observed with respect to weather conditions [32]. The dominance of some Mucorales species, such as *Apophysomyces*, in Indian soil may further support this hypothesis [73]. The use of cow dung for therapeutic use is also suspected to be a potential factor since animal manure may act as a source of infection [74,75]. Other potential factors that can increase the risk of CAM are shown in Fig 1, while Box 1 lists possible measures that can alleviate the potential risk of CAM.

**Box 1. Possible precautionary steps to prevent COVID-19–associated mucormycosis [14,20,42,68,74,76]**

**Treatment and management**

- Making early disease diagnosis
- Not missing early signs
- Optimal and judicious use of systemic corticosteroids
- Rationale use of antibiotics
- Supervised use of drugs that may increase infection risk
- Maintaining glycaemic control
- Classification of possible, probable, and proven infection
- Segregating patients based on COVID-19 disease status
- Timely therapy initiation

**Hospital environment and advisory bodies**

- Ensuring quality control of oxygen supply
- Proper sanitisation of oxygen cylinders
- Preserving a hygienic hospital atmosphere
- Using disposable oxygen humidifiers
- Using clean distilled water in oxygen humidifiers and concentrators
- Following better risk messaging strategies
- Proper use of medical checklists (like Mucor)
- Increasing the number of testing facilities
- Increasing mass urine testing for diabetes

**Personal safety**

- Maintaining personal hygiene during and post-COVID-19
- Increasing awareness during hospital discharge after recovery from COVID-19
- Avoiding self-medication and panic-driven practices
- Judicious use of social media for attaining health information
- Barrier mask covering the nose and mouth
- Get vaccinated for COVID-19

## What do the treatment guidelines recommend?

As of July 19, 2021, at least 11 different India-specific guidance documents or CAM directives are available (Table 1). They mostly follow the 2019 global guidance on mucormycosis by the European Confederation of Medical Mycology (ECMM) and Mycoses Study Group Education and Research Consortium [77]. Code Mucor, the most comprehensive among them, proposes a simple but reliable staging system for CAROCM and a treatment algorithm that suits each substage [76]. In general, most national guidelines warn about controlling diabetes and optimising corticosteroids use. Immediate and extensive radiology-guided surgical intervention is the treatment of choice. Orbital exenteration is suggested for patients in CAROCM stage 3a-c with the worsening of the orbital component within 72 hours. In advanced disease conditions (stage 4), aggressive debridement of the paranasal sinuses with or without turbinectomy or palatal resection or orbital wall resection may be necessary [76]. The India-centric guideline by ECMM and the International Society for Human and Animal Mycology (ISHAM) also advises extensive debridement of external infected tissues, including bones. It suggests repeated procedures in case of recurrence [78]. In the largest sample of 2,826 CAROCM patients, surgery decreased (from 67% to 39%) mortality and disease progression. Moreover, surgical intervention seems not to be contraindicated to CAROCM patients with CNS involvement, as orbital exenteration in all patients with stage 4 disease has shown stable residual or regressed lesions with improved survival after 3 weeks [34]. The Indian Medical Association provides a long list of suggested surgical procedures, including maxillectomy, depending on the need of affected patients [79].

**Table 1:** Recommendations on treating CAM in India.

S. No.	Recommending Guidelines	Surgery	Antifungal Treatment	Follow-up, Monitoring, and Other Advisory
1	All India Institute of Medical Sciences [42]	•Debridement after stabilising condition and ensuring postoperative care, including ventilator support	•Amphotericin B •Posaconazole	•Twice-daily evaluation of mucor checklist
2	Clinical Infectious Disease Society [35]	•Extensive surgical debridement	•Amphotericin-B deoxycholate ○1 mg/kg/day IV. ○4–6 weeks •Liposomal Amphotericin B ○5 mg/kg/day in 5% dextrose ○4–6 weeks •Isavuconazole IV/Tab ○200 mg thrice daily for 2 days ○Followed by 200 mg once daily ○4–6 weeks •Posaconazole IV/Tab ○300 mg twice daily Day 1 ○Followed by 300 mg once daily ○4–6 weeks	•1 L 0.9% saline to be infused with 20 mEq potassium chloride over 2 hours before each dose of Amphotericin B. •Premedication: paracetamol, diphenhydramine 30 minutes before infusion •Reconstituted Amphotericin B deoxycholate is to be diluted in 5% distilled water to obtain a concentration of 0.1 mg/ml and infused over 4 hours •Potassium is to be replaced if there is hypokalemia •Hypomagnesemia should be checked in refractory hypokalemia •If creatinine increases >2 times baseline, amphotericin therapy should be temporarily stopped and restarted after it returns to normal
3	Code Mucor [76]	•Surgery should be prioritised for all proven CAROCM patients •Endoscopy and magnetic resonance imaging/computed tomography-guided debridement for stages 1–2, 3a-b •For stage 3a-c: Orbital exenteration •Additionally, for stage 4a-b: orbital exenteration + aggressive debridement of paranasal sinuses (± turbinectomy ± palatal resection ± orbital wall resection) •For stage 4c-d: Surgery (as that of stage 3a-c) is suggested if systemic condition permits	•Immediate induction therapy ○Liposomal Amphotericin B (5–10 mg/kg BW) with strict metabolic control ○Deoxycholate or Lipid complex are alternatives ○If Amphotericin B is contraindicated: ▪Isavuconazole IV •200 mg thrice daily for first 2 days •200 mg once daily from day 3 ▪Posaconazole IV •300 mg twice daily on day 1 •300 mg once daily from day 2 •Continue induction therapy for at least 4 weeks •IV Amphotericin B (5–10 mg/kg BW) •Step down therapy for 3–6 months: ▪Isavuconazole oral •200 mg thrice daily on first 2 days •200 mg once daily from day 3 ▪Posaconazole oral •300 mg twice daily on day 1 •300 mg once daily from day 2	•For stage 4c-d: Supportive treatment can be considered only if surgery is not feasible •Step down therapy is recommended for at least 6 weeks following clinical regression and radiological regression or stabilisation •If refractory (after step down therapy), ECMM/MSG-ERC guidelines for salvage therapy is suggested.
4	Directorate General of Health Services–India [80]	•Surgical debridement	•Preferred: Liposomal Amphotericin-B •5 mg/kg in 5% or 10% dextrose •High dose (10 mg/kg), if the CNS is involved •Step down therapy: ▪Tab. Posaconazole •Delayed-release •300 mg twice daily on day 1 •Followed by 300 mg daily ▪Tab. Isavuconazole •200 mg thrice daily on days 1 and 2 •Followed by 200 mg daily	•Treatment to be continued until resolution of signs and symptoms •Conventional Amphotericin B (deoxycholate; 1–1.5 mg/kg) can be tried if the liposomal form is unavailable.
5	European Confederation of Medical Mycology and the International Society for Human and Animal Mycology [78]	•Early and aggressive surgical resection and debridement of affected tissues •CAROCM infection: complete debridement of the external infected tissues, including bones and internal tissues by endoscopic debridement or orbital exenteration •If recurrent: repeated resection and debridement •Pulmonary mucormycosis: Resection of the affected lung (if localised or single lobe involvement)	•First line: Early state of optimally lipid-based formulations of Amphotericin B •Liposomal Amphotericin B is strongly recommended: •5 mg/kg/day in 200 ml 5% dextrose •2–3 hours infusion •For 3–6 weeks •Among azoles, posaconazole and isavuconazole are effective •Itraconazole is generally not recommended; if other treatments are unavailable, it can be considered: •200 mg/kg/day •For 3–6 weeks •To be taken with acidic beverages like cola •Iron chelators (deferasirox) •Considered in patients with diabetes •To be avoided in haematological malignancy	•Itraconazole: •Concomitant use of proton-pump inhibitors decreases the drug absorption •Therapeutic monitoring is required after 5 days of treatment
6	Fungal Infection Study Forum [81]	•Extensive debridement •If eye involved: exenteration of eye •In pulmonary, if the lesion is localised or in one lobe	•Preferred: Liposomal Amphotericin-B •5 mg/kg/day in 200 cc 5% dextrose •2–3 hours infusion •High dose (10 mg/kg/day), if the brain is involved •Amphotericin B deoxycholate: •1 mg/kg/day in 5% dextrose •6–8 hours infusion •Premedication is required to avoid infusion reaction •For patients intolerant to Amphotericin B: ▪Tab. Posaconazole •300 mg twice daily on day 1 •Followed by 300 mg once daily ▪Tab. Isavuconazole •200 mg thrice daily on days 1 and 2 •Followed by 200 mg once a day •If polyene or the 2 azoles are not available: ▪Itraconazole •200 mg thrice daily for 3–6 weeks with food •Order of dosage form preference: injection, suspension, tablet	•No antifungal prophylaxis required •For posaconazole therapy, trough level at 1 week to be checked and interacting drugs to be avoided •Clinical and radio-imaging monitoring for response and detecting disease progression •After 3–6 weeks of amphotericin B therapy, consolidation therapy (posaconazole/isavuconazole) for 3–6 months •For itraconazzole, therapeutic drug monitoring after 5 days and weekly liver function tests are recommended
7	Government of Kerala, Health & Family Welfare Department [82]	•Extensive debridement •If eye involved: exenteration of eye after consultation with ophthalmologist	•Preferred: Liposomal Amphotericin-B •5 mg/kg/day in 200 cc 5% dextrose •2–3 hours infusion •High dose (10 mg/kg/day), if brain is involved •Amphotericin B deoxycholate: •1 mg/kg/day in 5% dextrose •6–8 hours infusion at the rate of 0.08 mg/kg/hour •Premedication is required to avoid infusion reaction •For patients intolerant to Amphotericin B: ▪Tab/IV Posaconazole •300 mg twice daily on day 1 •Followed by 300 mg once daily ▪Tab/IV Isavuconazole •200 mg thrice daily on days 1 and 2 •Followed by 200 mg once a day	•Preloading with 1 L normal saline in patients without risk of fluid overload and administering 1 L normal saline after infusing Amphotericin B will help limit nephrotoxicity. •Renal function and potassium level to be monitored while treating Amphotericin B. •If hypokalemia is not resolved with IV potassium chloride, hypomagnesemia should be ruled out. •For posaconazole therapy, trough level at 1 week to be checked and interacting drugs to be avoided •After 3–6 weeks of amphotericin B therapy, consolidation therapy (posaconazole/isavuconazole) for 3–6 months
8	Indian Council for Medical Research [43]	•Extensive debridement	•Amphotericin B with prior normal saline IV infusion •Antifungal therapy for a minimum of 4–6 weeks	•Clinical and radio-imaging monitoring for response and detecting disease progression •No antifungal prophylaxis required
9	Indian Medical Association [79]	•Extensive surgery •Endoscopic sinus surgery •Turbinectomy •Maxillectomy •Zygoma debridement •Debridement of orbital floor/walls with localised debridement •Exenteration of eye •Anterior table debridement •Posterior table cranialisation •Debridement of osteomyelitic skull bone	•First-line ▪Preferred: Liposomal Amphotericin-B •5 mg/kg/day in 200 cc 5% dextrose •2–3 hours infusion •High dose (7.5–10 mg/kg/day), if brain is involved ▪Inj. Amphotericin-B Deoxycholate •1 mg/kg/day in 5% dextrose •6–8 hours infusion •Premedication: Nonsteroidal anti-inflammatory drugs and/or diphenhydramine or acetaminophen with diphenhydramine or hydrocortisone, normal saline pre-infusion (500–1,000 ml) ▪Inj. Amphotericin-B Lipid Complex •5 mg/kg/day •Second-line (azoles) ▪Tab. Posaconazole •Delayed-release •300 mg twice daily on day 1 •Followed by 300 mg once daily with food ▪Tab. Isavuconazole •200 mg thrice daily on days 1 and 2 •Followed by 200 mg once a day	•Renal function and potassium level to be monitored for first-line treatment •For posaconazole therapy, trough level at 1 week to be checked and interacting drugs to be avoided •For isavuconazole therapy, clinical, radiological, and microbiological monitoring is suggested for checking treatment response and disease progression •For patients who are intolerant to Amphotericin B, azole therapy can be considered.
10	National Health Mission–Himachal Pradesh [83]	•Aggressive surgical debridement	•First-line ▪Inj. Amphotericin-B Deoxycholate •1–1.5 mg/kg/day •4–6 hours infusion •Premedication: Nonsteroidal anti-inflammatory drugs and/or diphenhydramine, or acetaminophen with diphenhydramine, or hydrocortisone, normal saline pre-infusion (500–1,000 ml) ▪Liposomal Amphotericin-B •5 mg/kg/day •High dose (7.5–10 mg/kg/day), if the CNS is involved ▪Inj. Amphotericin-B Lipid Complex •5 mg/kg/day •Second-line (azoles) ▪Tab/IV Isavuconazole •200 mg every 8 hours for the first 6 doses •Followed by 200 mg every 24 hours thereafter ▪Tab/IV Posaconazole •300 mg every 12 hours on day 1 •Followed by 300 mg every 24 hours •Oral dose to be taken with food •Combination therapy •Lipid polyenes + echinocandins (caspofungin, micafungin, anidulafungin) •Lipid polyenes + azole (posaconazole + isavuconazole) •Triple therapy (lipid polyene + echinocandin + azole)	•Dosing of Inj. Amphotericin-B deoxycholate: •Renal impairment: 50% reduction in total daily dose or dosing alternative days •Haemodialysis or continuous renal replacement therapy or hepatic impairment: no dose adjustment required •Renal function test and urine output to be monitored •IV posaconazole should be avoided in patients with moderate or severe renal impairment (creatinine clearance <50 mL/minute) due to the potential for accumulation of the betadex sulfobutyl ether sodium vehicle unless justified. •Larger studies are needed to establish whether combination therapy is beneficial. •Lipid polyene + echinocandin combination improves survival rate among disseminated mucormycosis, including CNS disease yield better outcome than monotherapy with polyenes.
11	World Health Organisation, South-East Asia: India [84]	•Surgical debridement	•Preferred: Liposomal Amphotericin-B •Posaconazole •Isavuconazole	-

CAM, COVID-19–associated mucormycosis; CAROCM, COVID-19–associated rhino-orbital-cerebral mucormycosis; CNS, central nervous system.

All guidelines recommend the use of systemic antifungal agents without any prophylactic therapy. The Indian Council of Medical Research (ICMR) recommends administering antifungal therapy for at least 4 to 6 weeks [43]. Slow infusion of liposomal amphotericin B (AmB, 5 mg/kg/day) is the preferred choice. A higher dose of up to 10 mg/kg/day is suggested if the infection spreads to the CNS [80]. Deoxycholate and lipid complex of AmB are considered alternatives [76]. Pyrexia, acute renal injury, hypokalaemia, increased levels of creatinine in blood, multiorgan dysfunction syndrome, and renal impairment are the leading adverse events (AEs) reported for AmB [85]. Hence, it is essential to assess renal function and potassium level during AmB treatment. In renal impairment, a dose reduction of up to 50% is recommended [83]. In patients contraindicated to AmB, selected azoles are suggested. Isavuconazole or posaconazole are the most preferred second-line drugs for salvage therapy. At the same time, itraconazole is recommended only by the Fungal Infection Study Forum and ECMM/ISHAM when AmB or the primary azoles are not available [78,81]. Therapeutic monitoring is recommended for itraconazole and posaconazole at 5 and 7 days of respective treatments. Mucormycosis in patients with neutropenia or graft-versus-host-disease may be managed with oral fluconazole; oral prophylaxis with itraconazole and voriconazole are suggested [28]. The dose regimens for all systemic anti-CAM agents indicated by different guidelines are detailed in Table 1, while the most common reported AEs are listed in Table 2.

**Table 2:** Common AEs of systemic fungal agents used to treat CAM as reported in the US Food and Drugs Administration Adverse Events Reporting System [85].

Antifungal drug*	Number of AEs^^^			Common AEs
	Total cases	Serious cases (including deaths)	Death cases	
AmB	10,958	10,163	4,235	Drug ineffective, off-label use, pyrexia, acute kidney injury, hypokalaemia, blood creatinine increased, multiple organ dysfunction syndrome, renal impairment, condition aggravated, renal failure, dyspnoea, chills, death
Isavuconazole	659	647	250	Death, product use in unapproved infection, off-label use, drug ineffective, drug interaction, nausea, diarrhoea, infection, febrile neutropenia, pneumonia, pyrexia, *Aspergillus* infection, acute kidney injury
Posaconazole	3,897	3,214	992	Drug interaction, drug ineffective, death, product use in unapproved infection, off-label use, pyrexia, pneumonia, *Aspergillus* infection, condition aggravated, neutropenia, AE, diarrhoea, acute kidney injury
Itraconazole	10,789	10,219	1,249	Drug interaction, dermatitis, pruritus, drug ineffective, pyrexia, dyspnoea, nausea, oedema peripheral, condition aggravated, hepatic function abnormal, asthenia, urticaria, rash maculo-papular

*Drugs in generic form.

^^^Data as of March 31, 2021.

AE, adverse event; amphotericin B; CAM, COVID-19–associated mucormycosis.

No guidance documents recommend combination therapy, citing the unavailability of supportive evidence, except one. The Himachal Pradesh National Health Mission suggests combining lipid polyenes with echinocandins (caspofungin) or azoles and having a triple regimen with all 3 drug classes included. However, it warns about the need for more extensive studies to prove these beneficial [83]. Calcineurin inhibitors tend to benefit antifungal resistance and virulence, with tacrolimus synergising posaconazole efficacy against *R*. *arrhizus* in vitro. Still, clinical effectiveness is not patent, mainly due to the emergence of various mutations and less use of immunosuppressants in these patients [71]. It is imperative to prevent potential drug–drug interactions of antifungals used to treat CAM when used in conjunction with experimental antiviral and immune therapies of COVID-19. Table 3 lists these interactions of specific anti-CAM agents recommended by various guidance documents against a few selected drugs currently used to treat COVID-19 [86]. All 3 guideline-suggested azoles may increase the colchicine and ruxolitinib exposure, while itraconazole-colchicine coadministration should be avoided. The use of AmB with steroids can cause hypokalemia, which requires dose adjustments. It is worthy of mentioning that the treatment choice for CAM is limited. This will create economic difficulties for patients in low-resource settings in countries like India [47].

**Table 3:** Potential drug–drug interactions of guidelines-suggested antifungal agents for treating mucormycosis with experimental COVID-19 antiviral or immune therapies [86].

Guidelines-suggested antifungal agent for treating mucormycosis	Experimental COVID-19 antiviral or immune therapy	Potential interaction	Required action
AmB	Dexamethasone	May cause hypokalaemia, which increases the risk of torsade de pointes	Dose adjustment or close monitoring
	Hydrocortisone		
	Methylprednisolone		
Isavuconazole	Colchicine	Increased colchicine exposure	
	Ruxolitinib	Increased ruxolitinib exposure	
Itraconazole	Budesonide	320% increased budesonide exposure	• Unlikely to be clinically relevant due to the short duration of inhaled budesonide used in COVID-19 treatment (2 weeks) • Monitor signs of systemic corticosteroid side effects
	Colchicine	Increased colchicine exposure	Coadministration not advised
	Ivermectin	• Increased ivermectin exposure (inhibition of CYP3A4) • Increased ivermectin transfer across the blood–brain barrier (inhibition of P-gp), resulting in higher concentrations in the brain and increased neurotoxicity	• Dose adjustment or close monitoring • Cautious use • Monitor for neurotoxicity
	Ruxolitinib	Increased ruxolitinib exposure	Dose adjustment or close monitoring
Posaconazole	Budesonide	Increased budesonide exposure	• Unlikely to be clinically relevant due to the short duration of inhaled budesonide used in COVID-19 treatment (2 weeks) • Monitor signs of systemic corticosteroid side effects
	Colchicine	Increased colchicine exposure	Coadministration not advised
	Ivermectin	Increased ivermectin exposure	Weak interaction; additional action unlikely required
	Ruxolitinib	Increased ruxolitinib exposure	Dose adjustment or close monitoring

AmB, amphotericin B; COVID-19, Coronavirus Disease 2019.

ICMR suggests forming a multidisciplinary team comprising microbiologist, internal medicine expert, intensivist, neurology specialist, otorhinolaryngologist, ophthalmologist, dentist, maxillofacial or plastic surgery specialist, and biochemist to manage patients with CAM [43]. All India Institute of Medical Sciences advises segregating patients based on COVID-19 status and suggests evaluating with mucor checklist twice daily [42]. The role of dental surgeons is imperative in the posttreatment phase as the patients may require intensive postoperative care and prosthetic rehabilitation [87,88]. An interesting and noteworthy study was reported from Fortis, a high standard private hospital in Mumbai, having an award-winning antimicrobial stewardship program [89]. During the country’s peak pandemic period (March 2020 to May 2021), 0 CAM cases were recorded among over 5,000 patients hospitalised for COVID-19 treatment. The reasons behind this laudable effort rely on following state government-specified COVID-19 treatment protocol, strict glycaemic control, optimal use of corticosteroids, and monoclonal antibodies.

## Future directions

Of the 16 mucormycosis studies registered on ClinicalTrails.gov, only 2 are related to CAM, of which an India-centric study is in the enrollment stage [90]. This project is expected to offer evidence on CAM features that currently wreak havoc in the country and contribute to the MUNCO registry. MUNCO is a recently launched international database of case reports on mycotic infections in COVID-19 [91]. The form that is used to register CAM cases contains key reporter (physician) information, patient details (without identifying information), case history related to COVID-19 and CAM, hospitalisation and treatment details, risk factors, COVID-19 vaccination, and impact of CAM on body systems [92]. Similarly, it would also be interesting to see how much CAM data goes into FungiScope, an established global registry for fungal infections [93]. This registry accepts case entry through ClinicalSurveys.net, while data can be searched on the FungiQuest retrieval page [94]. The other ClinicalTrials.gov study is from France. This completed study involved the characterisation of opportunistic fungal coinfections (including CAM) in mechanically ventilated patients with COVID-19 in ICU settings. As of September 03, 2020, 576 patients were enrolled, and the study results have not yet been published [95].

Novel therapies and treatment approaches are being explored to counter mucormycosis. The potential benefit of introducing statins into CAM therapy is recently proposed [96]. The authors suggest that statins may induce cytoprotective GRP78 expression, decrease infection risk, and synergise the anti-CAM drug levels in plasma. In vitro studies of statins showed positive results for various fungi; lovastatin could cause apoptosis of *Mucor racemosus*, while fluvastatin and rosuvastatin were beneficial against *Rhizomucor* and *Rhizopus* species. Interestingly, combination therapy of AmB + atorvastatin/lovastatin performed better against *R*. *arrhizus* than the efficacy produced by AmB alone [97]. This modality may also be clinically beneficial in high-risk patients with diabetes and hypercholesterolemia, but more extensive clinical trials are needed to prove its worthiness. Many other genetic and preclinical approaches are also being experimented. Mucorales peptides CotH3 and CotH7 have recently been identified to be associated with endothelial invasion of *Rhizopus* species. Anti-CotH3 antibodies are found to protect mice with neutropenia and DKA [98], while anti-integrin β1 antibodies restrict *Rhizopus delemar* attack of alveolar epithelial cells and guard mice with DKA from pulmonary mucormycosis [99]. Drugs under preclinical investigation that have the potential to become anti-Mucorale agents include VT-1161, SCH 42427, APX001A, colistin, and PC1244. Exploring novel treatment approaches, including the choice of using different routes for AmB administration (nebulised aerosols and topical application), is also being researched [100]. Interestingly, Indian researchers have come up with an innovative computer-aided customisable implant for the zygomatic portion of patients with mucormycosis. The implant, printed by metal laser sintering, could optimise the facial appearance and improve patient quality of life [101].

Immediate action should be taken to curtail COVID-19 and CAM, infections that currently ravage India, to prevent further spread. To achieve this, several fundamental steps, such as using the correct terminology to define the disease and transparency in reporting cases across regions, must be initiated. Since CAM is not transmitted between humans, no tracking like COVID-19 is required; however, the actual incidence would help plan appropriate health resource utilisation and prevention strategies. A further surge in CAM can be avoided by identifying high-risk groups and managing uncontrolled diabetes, which is the easiest to do among various potential risk factors. However, in India, since the large population lives in a rural area with many in poor resource settings, monitoring diabetes by testing blood sugar may not be a viable economical option. Therefore, the idea of Mass Urine Sugar Testing to Assess and Regulate Diabetes (MUSTARD) may come to the rescue. Urine-based testing is less costly (5 US cents), quick, and easy to perform among the masses [20]. Penultimately, mass vaccination is the stepping stone to achieving immunity to COVID-19, while mass surveillance is crucial to tackling CAM. As of July 15, 2021, 391 million doses of the COVID-19 vaccine were administered in the country [3]. Governmental agencies currently run massive campaigns to prevent vaccine hesitancy, and the results are promising as there is an increase in the number of vaccinated populations. Finally, it will be appropriate to observe the characteristics in Indian patients with “Long COVID” coinfected with CAM.

**Key learning points**

- Information on Coronavirus Disease 2019 (COVID-19)–associated mucormycosis (CAM) in India are evolving.
- Diabetes in COVID-19 induces immune changes, resulting in secondary infections.
- At least 11 different India-specific guidance documents/directives are available.
- Only 2 ClinicalTrials.gov studies are CAM-related; an Indian study is enrolling patients.
- Initiatives to vaccinate the population against COVID-19 are currently underway.

**Top five papers**

1. Sen M, Honavar SG, Bansal R, Sengupta S, Rao R, Kim U, et al. Epidemiology, clinical profile, management, and outcome of COVID-19-associated rhino-orbital-cerebral mucormycosis in 2826 patients in India—Collaborative OPAI-IJO Study on Mucormycosis in COVID-19 (COSMIC), Report 1. Indian J Ophthalmol. 2021;69(7):1670–1692. doi: 10.4103/ijo.IJO_1565_21
2. Patel A, Agarwal R, Rudramurthy SM, Shevkani M, Xess I, Sharma R, et al. Multicenter Epidemiologic Study of Coronavirus Disease-Associated Mucormycosis, India. Emerg Infect Dis. 2021;27(9). doi: 10.3201/eid2709.210934
3. Pal R, Singh B, Bhadada SK, Banerjee M, Bhogal RS, Hage N, et al. COVID-19-associated mucormycosis: An updated systematic review of literature. Mycoses. 2021. doi: 10.1111/myc.13338
4. Honavar SG. Code Mucor: Guidelines for the diagnosis, staging and management of rhino-orbito-cerebral mucormycosis in the setting of COVID-19. Indian J Ophthalmol. 2021;69(6):1361–1365. doi: 10.4103/ijo.IJO_1165_21
5. The Lancet. India’s COVID-19 emergency. Lancet. 2021;397(10286):1683. doi: 10.1016/s0140-6736(21)01052-7
